# A lab-on-chip platform for simultaneous culture and electrochemical detection of bacteria

**DOI:** 10.1016/j.isci.2022.105388

**Published:** 2022-10-19

**Authors:** Sangam Srikanth, U.S. Jayapiriya, Satish Kumar Dubey, Arshad Javed, Sanket Goel

**Affiliations:** 1 Department of Mechanical Engineering, Birla Institute of Technology and Science (BITS) Pilani, Hyderabad Campus, Hyderabad 500078, India; 2 Department of Electrical and Electronics Engineering, Birla Institute of Technology and Science (BITS) Pilani, Hyderabad Campus, Hyderabad 500078, India; 3 MEMS, Microfluidics and Nanoelectronics (MMNE) Lab, Birla Institute of Technology and Science (BITS) Pilani, Hyderabad Campus, Hyderabad 500078, India

**Keywords:** Bio-electrochemistry, Applied sciences, Sensor system

## Abstract

A simple, cost-effective and miniaturized lab-on-a-chip platform has been developed amenable to perform simultaneous cultivation and detection of bacteria. A microfluidic chamber was integrated to screen-printed electrodes for electrochemical detection of bacteria. The temperature required for the bacterial culture was provided through the optimized laser-induced graphene heaters. The concentration of bacteria was quantified accurately with the three-electrode system in the range of 2 × 10^4^ to 1.1 × 10^9^ CFU/mL without any need of biological modifications to the electrodes. The viability of cultured bacteria in the microfluidic device was also confirmed through fluorescent imaging. Furthermore, the metabolic activity of the cultured bacteria was validated through a miniaturized microbial fuel cell. Furthermore, the specificity of electrodes was also performed through electrochemical technique. Finally, a handheld and portable lab-on-a-chip platform was realized by 3D packaging, integrated with a portable potentiostat for real-time and on-field applications.

## Highlights

• A miniaturized platform was developed to culture and detect bacteria simultaneously
• Bacteria was quantified with carbon electrodes without any biological modification
• Capability of quantifying bacterial growth in range of 2 × 10^4^ to 1.1 × 10^9^ CFU/mL
• A handheld and portable lab-on-a-chip platform was realized by 3D packaging

## Graphical abstract

**Figure:**
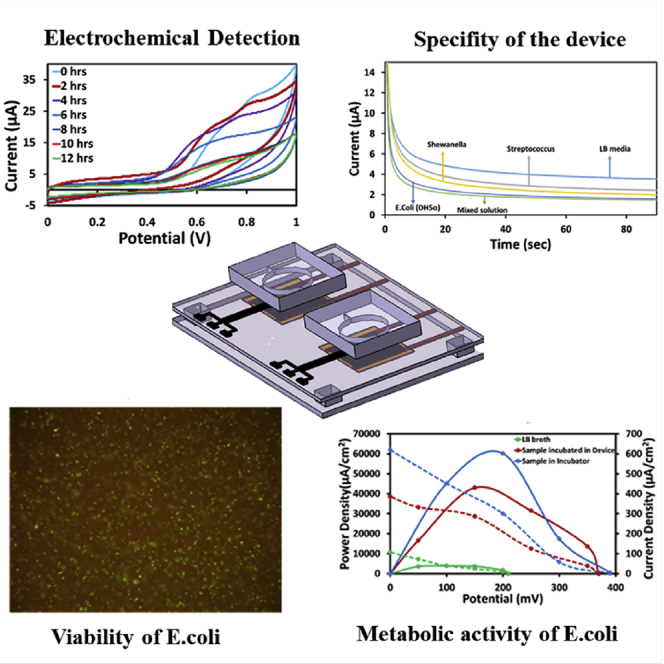

## Introduction

Detection of colony formation units (CFUs) of bacteria is essential in several fields such as medical investigations, food safety, and biotechnology (Xue et al., 2009). In general, traditionally, for any such bacterial detection, bacteria are cultured in a conventional bulky incubator at a specific temperature to accommodate their growth. After culture, the bacterial growth is determined in two ways: (1) determining the optical density using a spectrophotometer at a specified wavelength, usually at 600 nm and (2) determination of CFU per milliliter (CFU/mL) in a cultured sample i.e., the true concentration of bacterial growth using plate counting method. The first method does not give the exact count of the bacterial cells in the given solution, whereas the second method, although reliable, established, and accurate, is a time and labor invasive method. In recent times, many innovative techniques came into existence such as enzyme-linked immunoassay (ELISA) (Shan et al., 2016), polymerase chain reactions (Meng et al., 1996), cell detection through diffraction, flow cytometry, cell counters, fluorescence detection (Wildeboer et al., 2010; Zhu et al., 2012), surface plasma resonance (Si et al., 2011), microwave ring resonator (Narang et al., 2018), and electrochemical detection techniques (Sun et al., 2019). Although these methods are accurate and sensitive, factors such as high cost, time consumption, and requirement of personnel with expertise hinder their usability for on-field analysis. To overcome these challenges, a unique, simple, inexpensive, and an efficient method for detection of bacteria is required.

One such method is the implementation of electrochemical techniques, which is amenable to be integrated with bacterial culture. As summarized in Table 1, recently, electrochemical detection is gaining huge importance because of advantages such as faster analysis times, high sensitivity, and its minimum sample requirements (Kempler et al., 2021; Li et al., 2012). For instance, an electrochemical sensor integrated with a microfluidic system was recently reported, which can detect the concentration of bacteria ranging from 0.9 × 10^4^ CFU/mL (Altintas et al., 2018). This method implemented expensive gold electrodes with a complicated modification process with enzymes that are very sensitive to temperature, limiting its usage in practical applications. In another similar work, screen-printed carbon electrodes were modified with gold nanoparticles and are immobilized with enzymes such as horseradish peroxidase (HRP) and tetramethyl benzadine (TMB) in order to sense *E. coli* (*Escherichia coli*) bacteria in the range of 4 × 10^4^ to 4 CFU/mL (Shoaie et al., 2018). Although the device is sensitive, a tedious and lengthy modification process of the electrodes is required that holds it back for its utilization in real-time applications. In addition, gold electrodes were also utilized for detection of *E. coli* after modification with anti-*E. coli* antibodies for sensing in the range of 4.2 × 10^2^ CFU/mL to 4.2 × 10^5^ CFU/mL (Li et al., 2012). In another work, electrochemical technique was implemented to detect *E. coli* using oxidation reaction of nickel on a rotating disc type electrode. It was reported that the sensor was capable of rapid detection of bacteria in the order of 10^4^ CFU/mL (Ramanujam et al., 2021). Silver nanoparticles (AgNP) were also utilized for direct detection of *E. coli*. The established phenomenon that adherence of AgNP to bacterial cells results in increase of current when maintained at a suitable potential was utilized to detect the concentration of bacteria as a proof of concept (Sepunaru et al., 2015). Alternately, gold electroplated interdigitated electrodes were modified with anti-*E. coli* antibodies and were utilized for sensing *E. coli* through electrochemical impedance spectroscopy (EIS) (Ghosh Dastider et al., 2018). A similar work, where gold electrodes were immobilized with *E. coli* using antibody-binding method was reported to detect bacteria of concentration up to 10^5^ CFU/mL (Xu et al., 2020).

**Table 1:** Types of electrode materials, modification, and detection techniques with detection range reported by various works

Electrode material	Modification	Electrochemical technique	Detection range	Bulk/Miniaturized	References
ITO/Platinum/Ag/AgCl	*p*-Benzoquinone (BQ)	CV/Colorimetric	1.0× 10^3^ to 1.0 × 10^9^ cfu/mL	Bulk	Jayapiriya and Goel (2021)
Gold	Chitosan/MWCNT/GNP/anti *E. coli* antibody	CA	4.12 × 10^2^– 4.12 × 10^5^ cfu/mL	Bulk	Kaushik et al. (2017)
Gold	AuNP/HRP/anti- *E. coli* antibody/	CV/CA	0.99 × 10^4^–3.98 × 10^9^ cfu/mL	Miniaturized	Kempler et al. (2021)
Screen printed carbon electrodes	PANI/GNP/HRP/TMB	CV	4–4 × 10^6^ cfu/mL	Bulk	Kothuru et al. (2020)
Nickel/Platinum	Oxidation of nickel hydroxide (Ni(OH)_2_) to nickel oxyhydroxide (NiOOH)	CA	10^4^–10^10^ cfu/mL	Bulk	Li et al. (2012)
Carbon fiber/Calomel/graphite rod	Adherence of AgNP with *E. coli*	CV/CA	—	Bulk	Meng et al. (1996)
Gold	Anti *E. coli*/antibodies	EIS	39 cfu/mL in 2 h	Miniaturized	Narang et al. (2018)
Gold	Anti *E. coli*/antibodies	EIS	10^5^ cfu/mL	Bulk	Qin et al. (2010)
Screen printed carbon electrodes	MWCNT	CV/CA	2 × 10^4^ to 1.1 × 10^9^ cfu/mL	Miniaturized	Present work

It is evident from the available literature and recent attempts that in most of the work attempted at microfluidic platform, electrochemical sensing approach was utilized for bacterial samples. However, for detection in the above reported works, the samples were already cultured in a conventional platform to the required concentration level and then electrochemical analysis was performed on a bulk platform. This leads to a lack of the understanding of the growth of the bacterial concentration with time. Hence, there is a need for a simple, low-cost, reliable, effective, and a handy device that is capable of performing incubation and simultaneous detection of the growth of bacteria instantaneously. In order to achieve this, present work is focused in the direction to realize how to miniaturize the conventional incubation and detection platforms into a single handheld device. Earlier reported works (Zhou et al., 2019) suggest that culture of bacteria can be effectively done in (1) microchannels (Balagaddé et al., 2005; Chen et al., 2010), (2) microchambers (Held et al., 2010; Wu et al., 2015), and (3) microdroplets (Ho et al., 2020; Kaushik et al., 2017; Watterson et al., 2020). In addition, it was also reported that the reduced sample volumes used in microfluidic devices minimize the time taken for bacteria culture compared with the conventional incubator (Kaushik et al., 2017). Although bacterial culture was implemented in microfluidic channels, simultaneous detection of the bacterial growth was not monitored through electrochemical detection techniques, to the best of our knowledge.

**Figure 1:**
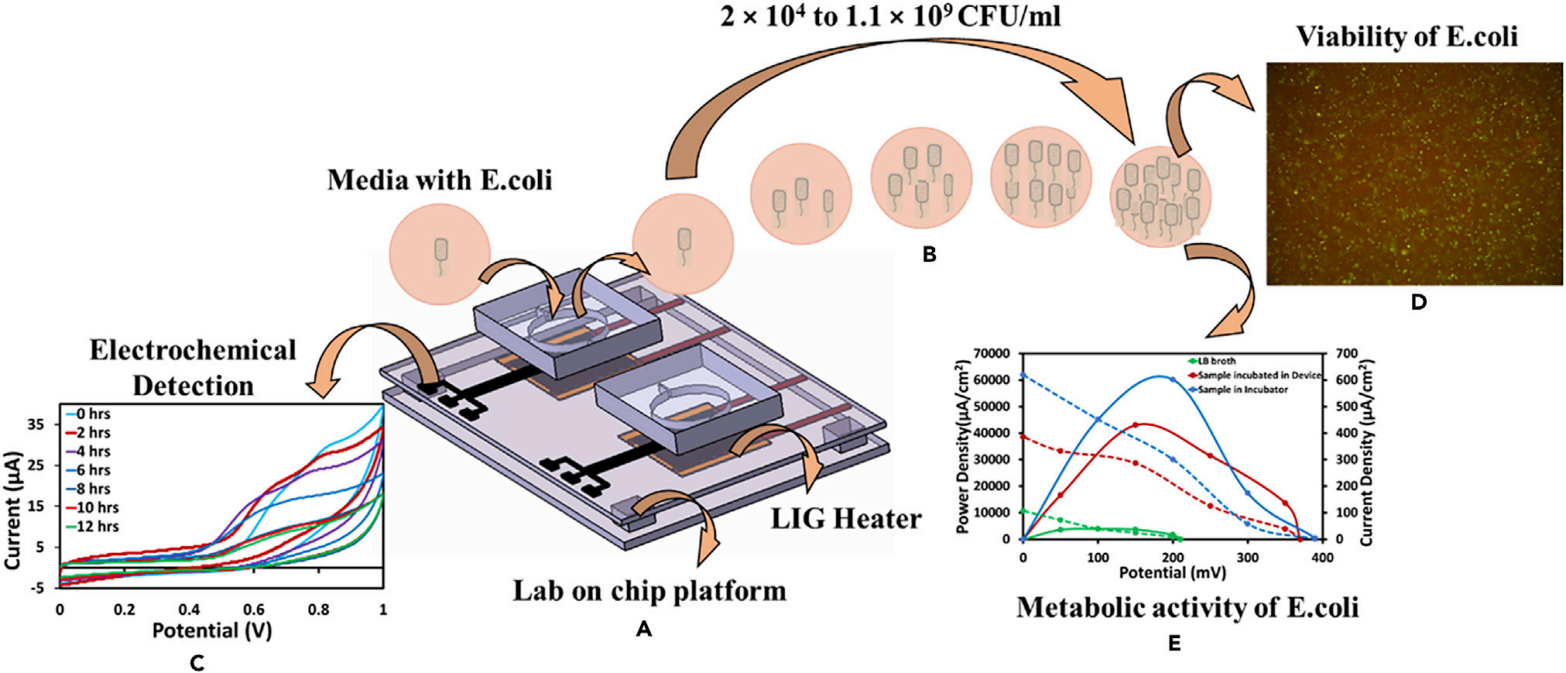
Schematic describing the overview of the presented work (A) microfluidics device integrated with screen printed electrodes and LIG heater; (B) on-chip incubation of bacteria; (C) simultaneous electrochemical detection of bacteria; (D) confirmation of viability through fluorescence imaging; (E) validation of metabolic activity by employing the cultured bacteria for microbial fuel cell (graph represents the polarization curves).

Motivated with such gaps and requirements, in this work, screen-printed carbon electrodes were fabricated and integrated to a microfluidic chamber. The integrated device (shown in Figure 1) is provided with the required temperature for bacterial growth and the timely growth of the bacteria was recorded electrochemically. In addition, the viability of the cultured bacteria was validated through fluorescence imaging. Furthermore, the metabolic activity of the cultured bacteria was corroborated with an enzymatic biofuel cell. Finally, a handheld device was realized and integrated with a portable potentiostat, enabling the usage of the device for on-field applications.

## Results and discussions

### Electrochemical detection of bacteria

In order to comprehend the growth of bacteria in the microfluidic device, two types of electrochemical detection techniques were performed: (1) cyclic voltammetry and (2) chrono amperometry. The peak currents in both the techniques were observed, and calibration plots were plotted through which the growth of bacteria is related in terms of current obtained in the detection technique due to the enzymatic activity of *E. coli*. In order to achieve this, 7 samples of known concentrations were chosen (2 × 10^4^–1.1 × 10^9^ CFU/mL). In brief, one sample of bacteria with 2 × 10^4^ CFU/mL was infused into the microfluidic device and another sample of same concentration was kept in a conventional incubator with orbital shaker at 36°C. Simultaneously, the temperature was continuously provided for the microfluidic device through the LIG heater. Later, at concentration of 2 × 10^4^ CFU/mL, electrochemical analysis was performed for both the samples individually in two different devices. The time for the first reading, i.e., for concentration of 2 × 10^4^ CFU/mL, was set to be taken at zero hours and the readings were continued up to 12 h with an interval of 2 h. After electrochemical analysis, the sample in the device was allowed to culture in the device itself, whereas another sample was kept in the incubator for culturing in conventional way for further time-based analysis of bacterial growth in the two cases. After 2 h, electrochemical measurements were recorded for both the samples, i.e., sample incubated in conventional incubator and sample incubated in the device. In a similar fashion, experiments were repeated for a period of 12 h. After 12 h, no significant changes in the current in either of the electrochemical techniques were identified and hence within 12 h, 7 different concentrations were recorded.

### Cyclic voltammetry for detection of bacteria

**Figure 2:**
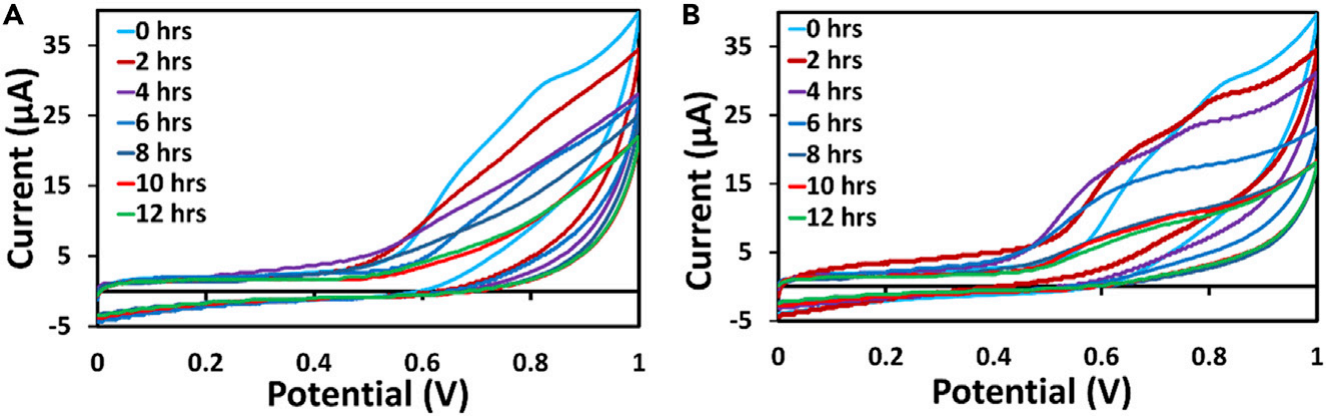
Electrochemical analysis of bacterial growth (A and B) Cyclic voltammograms recorded for 12 h corresponding to different concentrations of bacteria ranging from 2 × 10^4^ to 1.1 × 10^9^ CFU/mL in (A) conventional incubator and (B) microfluidic device.

In general, among all electrochemical techniques cyclic voltammetry gives a clear comprehension of transfer rate of electrons with the electrodes for a given analyte (Elgrishi et al., 2018). Few earlier works reported the implementation of CV for detection of bacterial concentration (Sun et al., 2019). In this context, initially cyclic voltammetry was performed to investigate the effect of growth in bacteria with time on the voltammograms. Cyclic voltammetry was performed for a period of 12 h in an interval of 2 h. Because the bacterial culture in a conventional incubator is a well-established protocol, initially, CV was carried out for the samples incubated conventionally, and these voltammograms were considered for comparison with that of the graphs obtained for sample in the microfluidic device. Figure 2 shows voltammograms performed for different samples at different time intervals. Figure 2A represents the cyclic voltammetric curve for bacteria sample cultured in the conventional way, and Figure 2B represents the curves obtained for culture performed in the microfluidic device. As can be seen from the Figure 2A, an oxidation peak current was observed at a potential of 0.8 V, and no reduction peak was observed. This potential of 0.8 V specifically corresponds to the peak of LB media as shown in Figure S2. With time and continuous provision of optimal temperature, it was very obvious that the concentration of the bacteria would be multiplying both in the microfluidic device or in the incubator. It can also be observed from the figure that the peak potential is gradually reducing with time similar to the works reported in literature (Sun et al., 2019).

**Figure 3:**
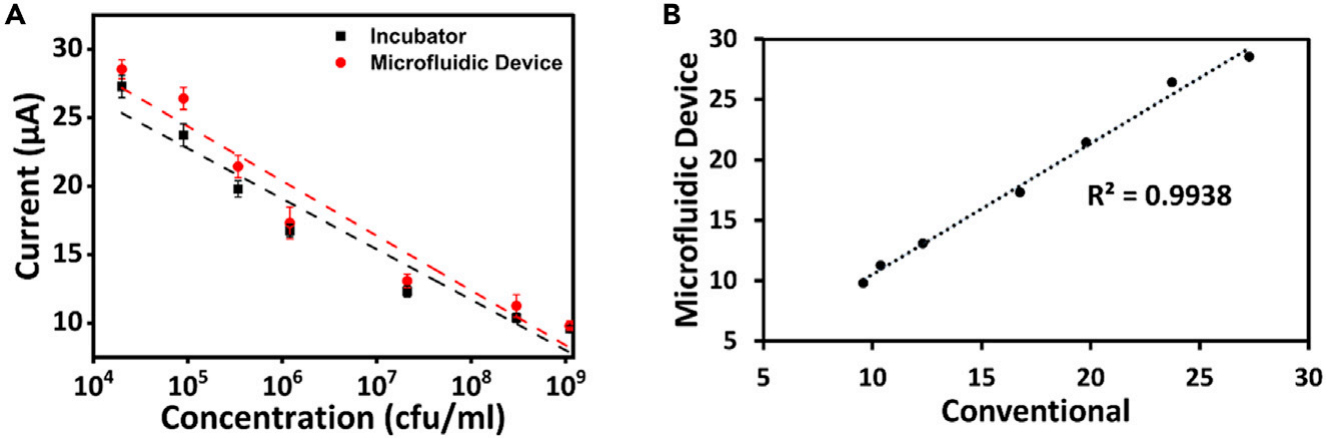
Plots derived from cyclic voltammograms (A) Calibration plots for sample incubated in conventional incubator and in microfluidic device (standard deviations are taken from three sets of experiments) taken from cyclic voltammograms. (B) comparative plot between the currents obtained from the conventional and microfluidic approach.

This implies that the peak of the LB is gradually decreasing, which must be possible only with the increase in the bacterial concentration in the media. The increase in concentration of bacteria reduces the amount of LB media, which corresponds to reduction of peak in the cyclic voltammogram. The similar trend can be seen in Figure 2B, for sample cultured in microfluidic device, which goes in line with the voltammograms obtained for conventionally cultured bacteria. In addition, the peaks obtained in Figure 2B are more distinguishable compared with that of Figure 2A. This can be related to the gradual increase of bacterial concentration in the microfluidic device. However, in order to comprehend the growth rate of bacterial concentration, a calibration plot has been considered for concentration against the observed peak currents from the CV graphs. It can be seen from Figure 3A that only minute difference in the currents was observed compared with the oxidation peak currents obtained in both the Figures 2A and 2B. The linear relationship function is identified as Equation 1 for microfluidic device with a regression of 0.94 and that of conventional approach was found to be Equation 2 with a regression of 0.95.

(Equation 1) Current(μA)=41.19−3.6(log10(ConcentrationinCFU/ml))

(Equation 2) Current(μA)=44.37−3.9(log10(ConcentrationinCFU/ml))

In addition, a comparison plot (Figure 3B) was considered to compare the conventionally incubated sample versus the microfluidic device wherein a linear fit was observed with a regression coefficient of 0.99. This suggests that the culture of bacteria on a microfluidic device is equally competent to that of the culture in a conventional incubator.

### Chronoamperometry for identification of bacterial growth

**Figure 4:**
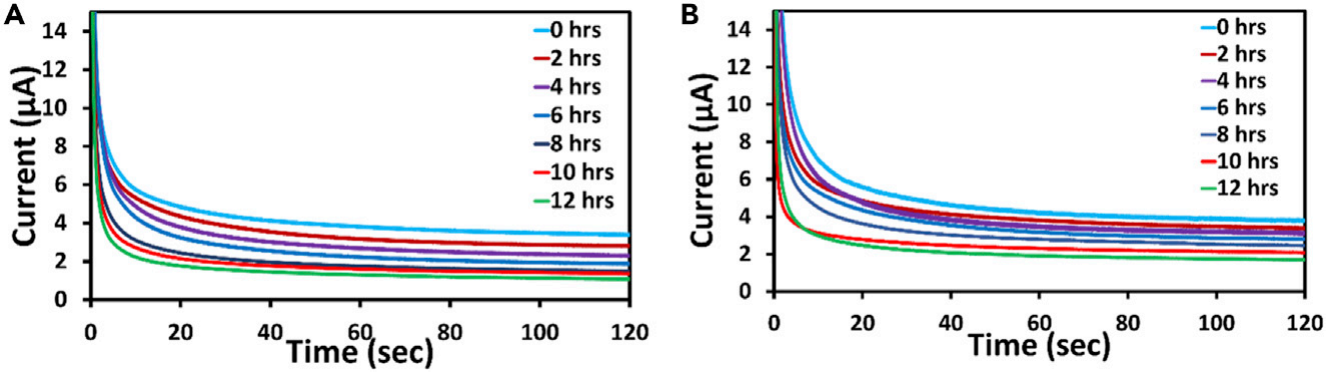
Electrochemical investigation of bacterial growth (A and B) Chronoamperometric curves recorded for 12 h corresponding to different concentrations of bacteria ranging from 2 × 10^4^ to 1.1 × 10^9^ CFU/mL in (A) conventional incubator and (B) microfluidic device.

In addition to cyclic voltammetry, chronoamperometry technique was also implemented to understand the nature of curves with increase in concentration of bacteria in conventional as well is in microfluidic device. In general, chronoamperometry provides the measure of current with respect to the time at a fixed potential. As per the CV graphs, because the peak was distinguished at around 0.8 V, the same potential was fixed for CA analysis. Each experiment was run at a fixed potential of 0.8 V for a span of 120 s. As can be seen from Figures 4A and 4B, there is a consistent decrease in the current with the increase of concentration similar to that was observed in the case of CV graphs. It can also be noted that after an initial decrement, the current stabilizes, and no further change in the current was observed. This suggests that a constant current is showcased for individual concentrations. Furthermore, the decrease in current values is observed in both the cases, i.e., conventionally cultures and that in the microfluidic device. This nature of curve suggests that the current gradually reduces with the increase in concentration of bacteria and this goes in line with the works reported earlier (Ramanujam et al., 2021).

**Figure 5:**
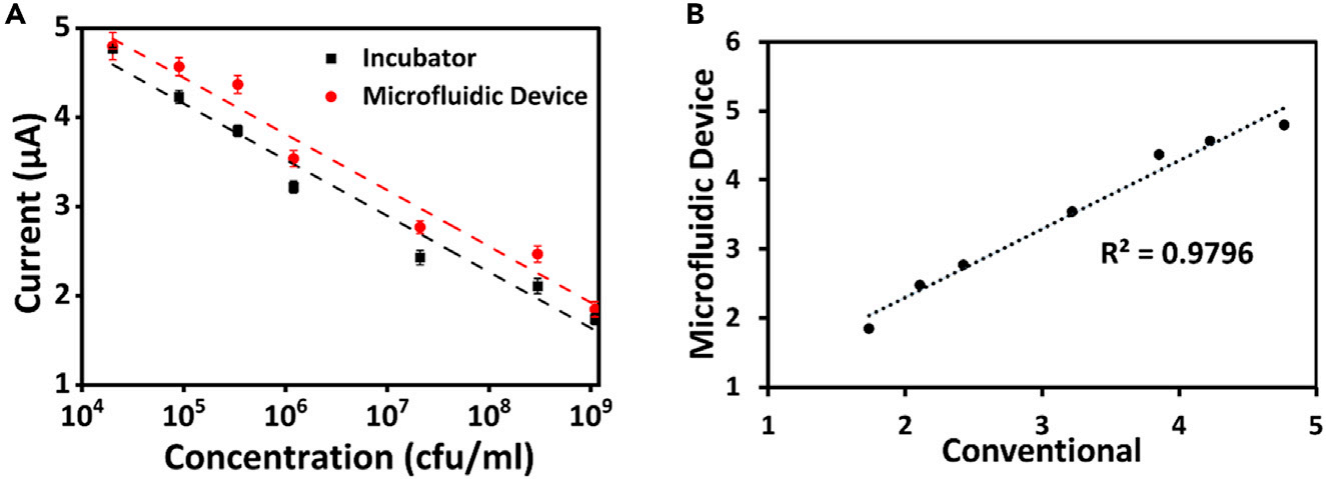
Plots derived from chronoamperometric curves (A) Calibration plots for sample incubated in conventional incubator and in microfluidic device (standard deviations are taken from three sets of experiments) taken from chronoamperometric curves; (B) comparative plot between the currents obtained from the conventional and microfluidic approach.

In addition, a calibration plot (Figure 5A) for concentration against current was plotted similar to the calibration plot of CV, whereby very minute changes in the current values were observed for both the cases. This calibration plot also helps in identifying the concentration of an unknown sample based on the current value within the limits of linear range 2 × 10^4^ to 1.1 × 10^9^ CFU/mL. The linear relationship followed the Equation 3 Current (μA) = 7.59–0.62(log_10_ (Concentration in CFU/mL) with a regression of 0.96 for microfluidic device and Equation 4 Current (μA) = 7.29–0.62(log_10_ (Concentration in CFU/mL) with a regression of 0.95 in the case of conventional approach.

(Equation 3) Current(μA)=7.59−0.62(log10(ConcentrationinCFU/ml))

(Equation 4) Current(μA)=7.29−0.62(log10(ConcentrationinCFU/ml))

Besides the calibration plot, a comparative graph (Figure 5B) for currents obtained with conventionally cultured bacteria against the current obtained with the microfluidic device was plotted, which exhibited a linear trend with a regression coefficient of 0.97. This imply that the culture performed in our microfluidic device is comparable with the one performed in a conventional apparatus.

### Fluorescent imaging for confirmation of live/dead bacterial cells

**Figure 6:**
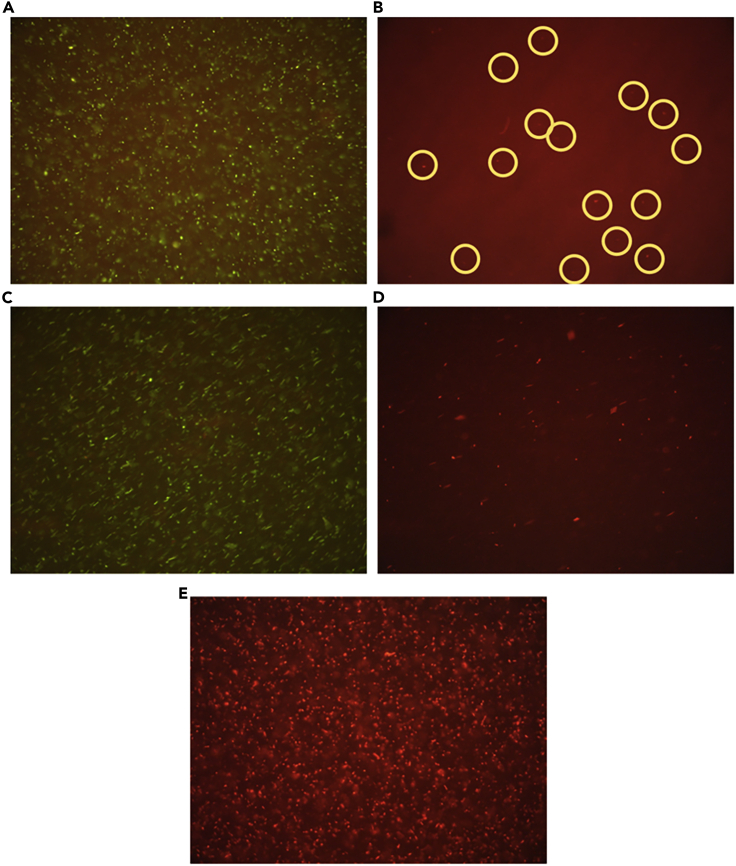
Viability studies of bacteria (A–E) Fluorescent images of viability of bacteria after 12 h culture for conditions as (A) live bacteria in conventional incubator; (B) dead bacteria in conventional approach; (C) live bacteria culture in microfluidic device; (D) dead bacteria in microfluidic device; and (E) completely dead bacteria detected in microfluidic device maintained at 50°C.

With the curves obtained from CV and CA, it can be analyzed that due to enzymatic activity, transfer of electrons is taking place and proper oxidation peak is being identified. However, to confirm that the decrease in peaks is owing to the growth of the bacteria, fluorescent imaging was performed to distinguish if the bacteria is alive or dead after the culture period. In order to achieve this, live/dead viability kit was utilized to identify dead and live bacteria. In brief, the viability kit includes two stains: (1) SYTO 9 and (2) propidium iodide, which gets binded to the live and dead bacterial cells, respectively. After 12 h of incubation in the conventional incubator and microfluidic device, the samples were taken out and were mixed with the bacterial stains and are left out in a dark place for 15 min. After the short incubation period, the samples were examined under fluorescence microscopy for three different conditions: (1) conventionally cultured bacteria, (2) bacteria cultured in the microfluidic device, and (3) sample in microfluidic device at 50°C. Figures 6A and 6B correspond to the condition of sample cultured conventionally, i.e., for the sample that is cultured in the incubator at 36°C in the presence of orbital shaker where it can be observed that very few bacterial cells were dead after incubation.

After 12 h, there was actively populated bacteria with very less number of dead bacterial cells. Figures 6C and 6D represent the sample cultured in microfluidic device wherein the growth of bacteria is equivalent to that of the conventionally cultured. However, the number of dead bacteria is a bit higher when compared with that of sample cultured in incubator. In another condition, bacteria were left in the microfluidic device, and a temperature of 50°C was constantly maintained. The fluorescent image of that sample showed that all the bacterial cells were dead (Figure 6E). Thus, the fluorescent images clearly showcase that there is definite growth in the microfluidic channel and the detection of their growth through the screen-printed electrodes is truly reliable.

### Corroboration for microbial fuel cell for metabolic activity

**Figure 7:**
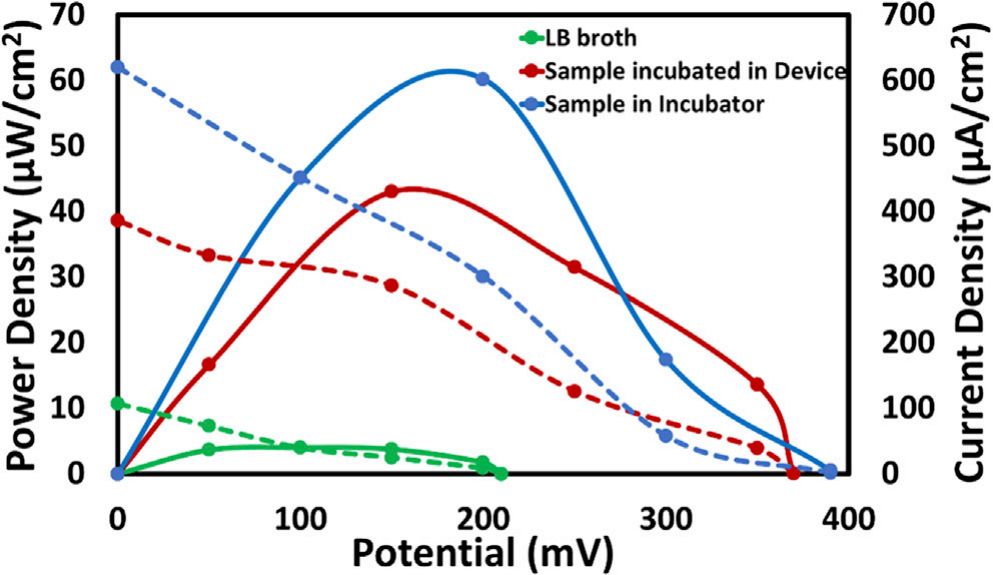
Polarization curves representing power density and current density for Plain LB media, bacteria cultured in incubator and bacteria cultured in microfluidic device

It is clearly evident from the fluorescent images that there was growth of bacteria with time in the microfluidic device and was simultaneously detected using a three-electrode system. However, besides the growth of bacteria, its metabolic activity is relatively important for its utilization for real-time applications. In this regard, to comprehend the metabolic activity of the cultured bacteria, a microbial fuel cell (MFC) was implemented. Earlier, few works reported the usage of bacteria as a fuel for running a fuel cell (Rahimnejad et al., 2015). So, as reported in one of our previous studies, a 3D-printed-paper-based microbial fuel cell was utilized to understand the effect of cultured bacteria over obtained power density (Jayapiriya and Goel, 2021). In brief, an anode and a cathode were fabricated using silver nano ink and carbon paste, respectively. A further enhancement of the cathode was done by modifying it with MnO_2_ nanoparticles. After fabrication of the electrodes, silver ink was used to provide electrical contacts. The performance of the microbial fuel cell, with three different samples (1) plain LB media (2) bacteria cultured in conventional setup, and (3) bacteria cultured in microfluidic device were considered to comprehend the efficiency of the bacterial fuel. Initially, 100 μL of LB media was drop-casted on the anode, and the readings were recorded once a stable open circuit voltage (OCV) was attained. The same was repeated for the other two cases. After the stabilization, the polarization curves were plotted to obtain maximum power and current density in all the three cases (Figure 7). In case of plain LB media, a maximum power density of 4 μW/cm^2^ was recorded, whereas the sample with bacteria delivered higher power output. The cultured bacteria using conventional setup generated a power density of 61 μW/cm^2^ and that of sample incubated in microfluidic device was found to be 43 μW/cm^2^. The reduced power density obtained from the sample cultured in microfluidic device can be attributed to the fact that more amount of dead bacterial cells was existing compared with the conventionally cultured bacteria as can be seen from Figure 6. However, these values of power densities confirm the successful growth of bacteria in the microfluidic device whose metabolism has produced quite a good amount of power density in comparison to other MFC available (Rewatkar et al., 2021). This suggests that the metabolic activity of bacteria cultured in the microfluidic device was relatively close to that of the sample cultured in conventional incubator.

### Realization of a handheld incubator cum detector for growth indication of bacteria

**Figure 8:**
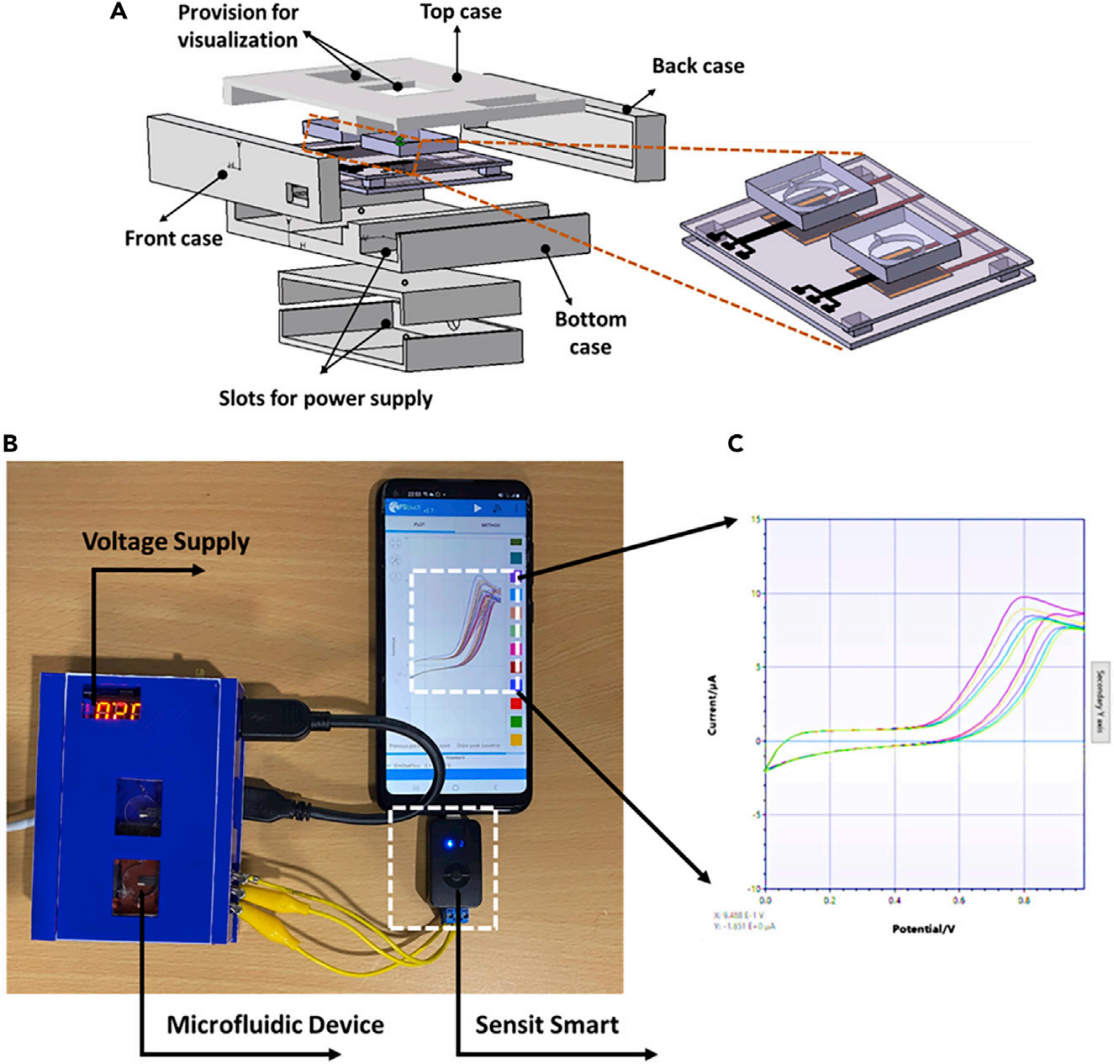
Development of portable device (A) schematic of the handheld device with different parts showcasing integrated microfluidic device. (B) Realized handheld device wherein the integrated microfluidic device is packaged, which is connected to a portable potentiostat (dimensions of the device being 110(l) ×85(b)×40(h) mm). (C) the output curves for bacterial growth in the microfluidic device.

In order to realize a miniaturized and an all-in-one platform, a 3D printed packaging was designed and realized to integrate the microfluidic device with the heater and the detection equipment (Figure 8). The voltage to the heater is supplied by a buck booster, and the electrochemical readings were taken using a handheld potentiostat (Sensit smart [PalmSens, Netherlands]) connected to a smartphone. Once the setup was ready, sample was infused into the microfluidic device, and the required voltage is supplied to the heater to provide sufficient temperature for the bacteria for incubation. Initial readings for plain LB media and the bacterial growth were shown in the inset of Figure 8.

### Specificity

**Figure 9:**
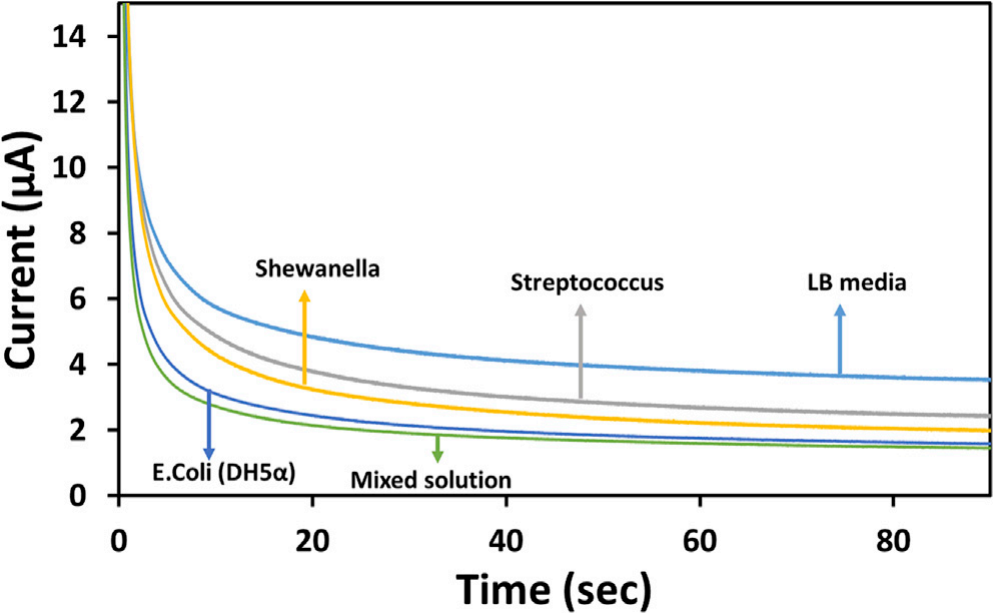
Chronoamperometric responses recorded for *E. coli*, Shewanella, Streptococcus, and mixed solution of the bacteria

In order to perform the specificity test on the fabricated device, two different bacteria namely Shewanella and Streptococcus were utilized. Initially, a blank sample of the media (Luria Bertani) was run and the current values were noted. Later on, cultured bacteria (*E. coli*, Shewanella, and Streptococcus) with almost a similar concentration of 2.1 × 10^9^ CFU/mL was considered for determining the current values using chronoamperometry technique (Figure 9). An initial run for blank resulted in higher values of current. As bacteria were introduced, there was significant changes in the values of current. It was identified that three different values of currents were recorded for the three bacteria but however, a mixture of all the three resulted in a current values similar to that of the *E. coli*, which is in line with the reported work (Ramanujam et al., 2021). This suggests that the current values in the mixed solution was nearly identical to that in the *E. coli*-only solution, confirming the sensor’s ability to detect signals from *E. coli* in particular.

### Conclusions

This work delves upon developing a miniaturized lab-on-a-chip platform, which was capable of simultaneously performing bacterial culture and detection of the bacterial concentration. Electrochemical techniques, such as cyclic voltammetry and chronoamperometry, were employed to investigate the growth of bacteria using screen printed electrodes. The device was capable of quantifying the bacterial growth within the range of 2 × 10^4^ to 1.1 × 10^9^ CFU/mL without any biological modifications to the electrodes. The viability and metabolic activity of the cultured bacteria were confirmed through fluorescence imaging and microbial fuel cell, respectively. 3D packaging was done to realize a handheld device integrated with a portable potentiostat expanding its usage in on field applications. In addition, specificity studies show that the device is capable to detect signals from *E. coli* in particular. These results pave a way to realize a miniaturized incubation cum detection platform that can be implemented for bacterial culture with greater advantages such as simple, reliable, quantifiable device with minimal sample volume, low cost and minimal power consumption. The future developments of this work would be to achieve lower detection limits and antibiotic susceptibility in microfluidic droplets in the integrated device. In addition, the device can also be modernized by integrating it with mobile-based fluorescence module for on chip viability indication. Furthermore, the integrated device can also be utilized for on-chip cell culture applications.

### Limitations of the study

The viability of the culture from the device can be further improved by modifying the device to have a shaking module that replicates the condition of the conventional incubator.

## STAR★Methods

### Key resources table

**Table:** 

REAGENT or RESOURCE	SOURCE	IDENTIFIER
**Chemicals, peptides and recombinant proteins**		

Conductive carbon ink	Engineered Materials system, Inc	CI-2001
Ag/AgCl	ALS Co. Ltd., Japan	011,464
Multi walled carbon nanotubes (MWCNT)	Sigma Aldrich	308068-56-6
Polyimide	Dali electronics	150FNB019
Potassium ferricyanide	AVRA Chemicals	13746-66-2
PDMS	Dow corning, USA	101697
Luria Bertani	SRL chemicals	29817

### Resource availability

#### Lead contact

Any information and requests for resources should be addressed to and will be responded by the lead contact, Sanket Goel (sgoel@hyderbad.bits-pilani.ac.in).

#### Materials availability

Our study did not generate any new unique reagents.

#### Data and code availability

• Data reported in this paper will be shared by the lead contact upon request.
• Our study did not report any unpublished custom code, software, or algorithm.
• Any additional information required to reanalyse the data reported in this paper is available from the lead contact upon request.

### Experimental model and subject details

This study does not use experimental methods typical in the life sciences.

### Method details

#### Materials

Conductive carbon ink was purchased from Engineered Materials system, Inc. Ag/AgCl was procured from ALS Co. Ltd., Japan. Multi walled carbon nanotubes (MWCNT) in powder form was procured from Sigma Aldrich, India. Polyimide sheet was purchased from Dali Electronics, India. A CO_2_ laser (VLS 3.60) was purchased from Universal Laser Systems, AZ, USA. Thermal camera was procured from FLUKE TECHNOLOGIES PVT. LTD., India. Potassium ferricyanide was procured from AVRA chemicals. PDMS was purchased from Dow corning, USA. Luria Bertani was purchased from SRL chemicals.

#### Methodology

##### Fabrication of screen printed electrodes

Screen printing technology has been chosen fabricate three electrode system over glass substrates. Initially, a mask of required dimensions has been prepared on a poly vinyl chloride (PVC) sheet using a commercial CO_2_ laser. The mask was adhered to the glass substrate. Later, a conductive carbon ink was laid over the mask and squeegee was used to spread the carbon ink over the required design uniformly. The substrate was then placed in an oven at 65°C for 1 h for complete drying. After drying, the PVC sheet was removed leaving the three electrode system on the glass substrate. In order to perform the electrochemical analysis, one of the three electrodes, to work as a reference electrode, was modified with Ag/AgCl ink and was kept in hot air oven at 65°C for a period of 15 min for drying.

Later, another electrode was modified with MWCNT and was treated as working electrode. The dimensions of the electrodes were found to be 1000 μm in width with a spacing of 350 μm between the two electrodes. The thickness of the screen-printed electrodes was found to be 50 μm. The surface area of the electrode for electrochemical reaction is around 0.2 mm^2^. The conductivity of the electrodes was identified as 3.06 × 10^3^ S/m, as reported in our previous work (Sangam et al., 2020).

##### Characterization of screen printed electrodes

In order to confirm the ability of the fabricated electrodes, first electrochemical detection of Ferricyanide was performed on the screen printed electrodes. Briefly, 5 mM of K_3_Fe(CN)_6_, an established mediating agent in combination with 1 mM of potassium chloride was mixed and was drop-casted on to the electrodes to comprehend the transfer rate. Cyclic voltammetry was performed at a scan rate of 50 mV/s which resulted proper transfer of electrons with a clear indication of oxidation and reduction peaks within potential of - 0.7 V to +0.7 V. In addition, the electrodes were characterized for plain LB media within a potential between 0 and 1 and a proper peak of the media was identified at a potential near to 0.8 V.

##### Fabrication of laser induce graphene heater

Laser induced graphene film was utilized as a heating source. The selection of power and speed, structural morphology and electrical and thermal characterization were described in detail in our previous work (Srikanth et al., 2021). In brief, a polyimide sheet of 250 μm thickness was adhered to a glass substrate and a CO_2_ laser of 10.6 μm wavelength was lased over the polyimide sheet to obtain laser induced graphene (Kothuru et al., 2020). The parameters used for laser scribing are 4.5 W power in combination with a speed of 1.375 mm/s scanning speed. The conductivity of the film obtained corresponding to these parameters was 11.29 × 10^2^ S/m. The dimensions of the effective heating area was 20 × 20 mm. Post laser scribing, the thickness of the film was observed to be 50 μm. Once the LIG film was fabricated, electrical contacts were provided using silver paste and copper tape. The fabricated film was then adhered to a glass substrate using double sided adhesive tape. The thermal characteristics were calibrated earlier and a potential of 2 V was applied to the LIG film which resulted the film to maintain a temperature of 36 ± 1°C. The temperature was constantly monitored using a thermal camera and a Pt100 thermal sensor.

##### Fabrication of microfluidic device and its integration

A microfluidic device was fabricated using soft lithography using PDMS. In brief, a design of the desired pattern was prepared in AutoCAD software. The pattern was cut on a PMMA (polymethylmethacrylate) using CO_2_ laser, adhered to a glass substrate and placed in a mold. As per the well-established protocol (Qin et al., 2010), PDMS was prepared by mixing the silicone elastomer with the curing agent in a ratio of 10:1 and is thoroughly mixed. The mixture was degassed in a desiccator and poured over the mold which was then placed in an oven at 65°C for curing. Post curing, the required holes were punched on the PDMS slab using blunt needles and was bonded to the glass slide with screen printed electrodes by treating the surfaces in the presence of oxygen plasma. The schematic of the final microfluidic device with the integrated electrodes. The microchannel can accommodate a volume of 300 μL of sample in it for experimentation.

##### Preparation of bacterial culture

Luria Bertani (LB) was prepared that typically contains 10 g/L of tryptone, 0.085 mol/L NaCl and 5 g/L yeast extracts. *E. coli* (DH5α) was cultured in LB media by inoculating 200 μL of bacteria in 20 mL of LB media. Post addition, proper mixing was done and the sample was kept in an incubator equipped with an orbital shaker set at 180 rpm and 36°C. Once the desired OD was reached, the sample was utilized for incubation. The colony formation units were detected using plating method.

### Quantification and statistical analysis

All the data was plotted and analyzed using Microsoft excel. For all the experiments where error bars were presented, the value of n is equal to 3.
