# Supplementary material for: A lab-on-chip platform for simultaneous culture and electrochemical detection of bacteria
Source: iScience. 2022 Oct 19;25(11):105388. doi: 10.1016/j.isci.2022.105388 (PMC9638781; doi:10.1016/j.isci.2022.105388)
Supplement: Document S1. Figures S1–S4 [file mmc1.pdf]

iScience, Volume *25*

Supplemental information

A lab-on-chip platform for simultaneous culture and electrochemical detection of bacteria

Sangam Srikanth, U.S. Jayapiriya, Satish Kumar Dubey, Arshad Javed, and Sanket Goel

List of Supplemental Information

Figure S1: Stepwise procedure indicating the fabrication of screen printed three electrode system, Related to Fabrication of screen printed Electrodes in STAR Methods

Figure S2: Cyclic voltammograms representing **(a)** Potassium Ferricyanide within a potential of -0.7 to + 0.7 and **(b)** Plain LB media in the absence of bacteria within a potential of 0 to 1, Related to Characterization of screen printed electrodes in STAR Methods

Figure S3: Thermal image of the microfluidic device showing the required temperature at the microfluidic chamber for an application of 2V to the LIG heater, Related to Fabrication of laser induce graphene heater in STAR Methods

Figure S4: Schematic of the microfluidic device integrated with screen printed electrodes and LIG heater, Related to Fabrication of microfluidic device and its integration in STAR Methods

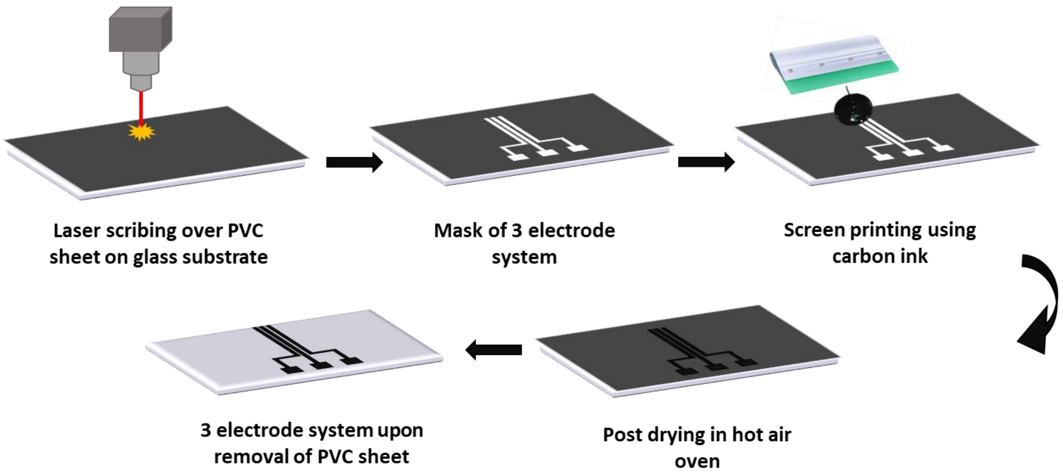

Figure S1: Stepwise procedure indicating the fabrication of screen printed three electrode system

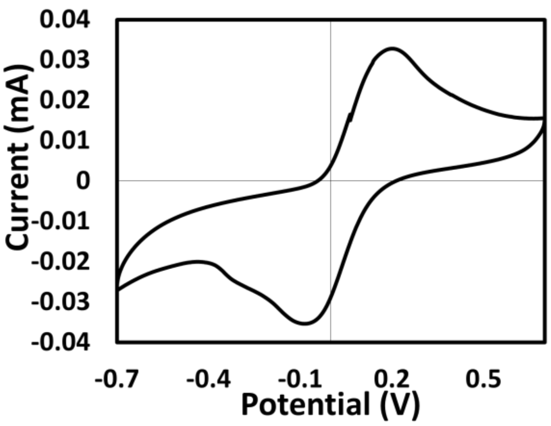

(a)

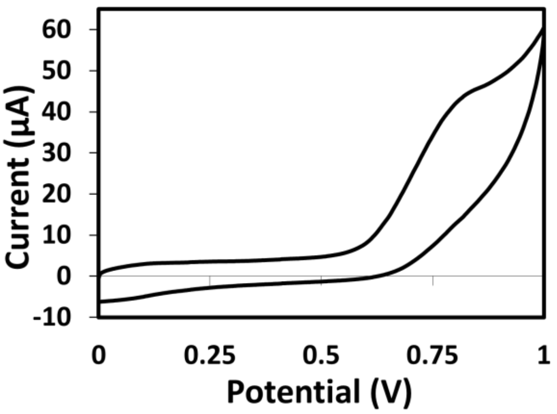

(b)

Figure S2: Cyclic voltammograms representing **(a)** Potassium Ferricyanide within a potential of -0.7 to + 0.7 and **(b)** Plain LB media in the absence of bacteria within a potential of 0 to 1

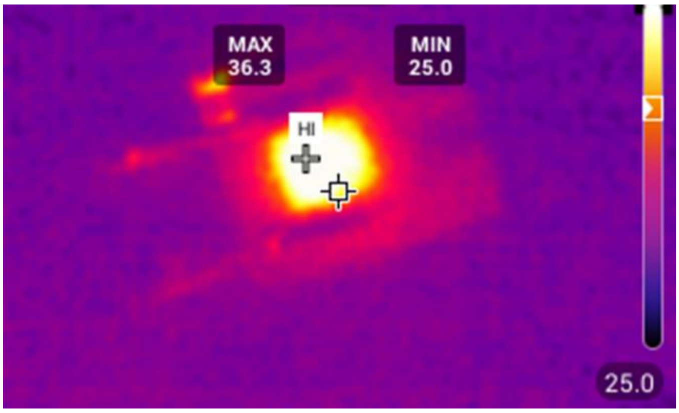

Figure S3: Thermal image of the microfluidic device showing the required temperature at the microfluidic chamber for an application of 2V to the LIG heater.

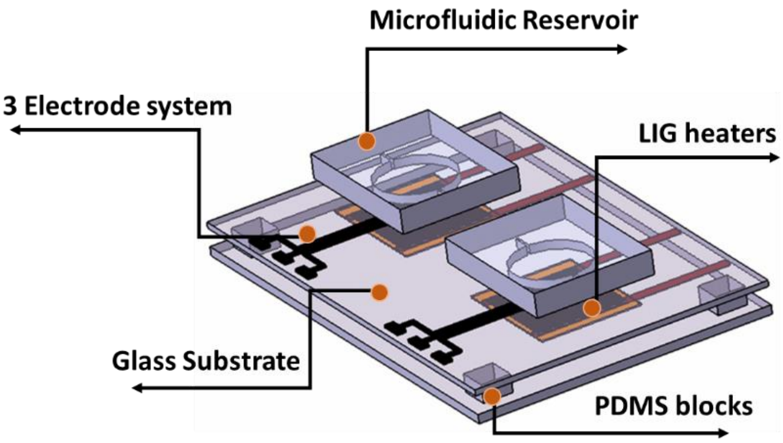

Figure S4: Schematic of the microfluidic device integrated with screen printed electrodes and LIG heater
